# Detecting peatland drains with Object Based Image Analysis and Geoeye-1 imagery

**DOI:** 10.1186/s13021-017-0075-z

**Published:** 2017-03-09

**Authors:** J. Connolly, N. M. Holden

**Affiliations:** 1 School of History and Geography, Dublin City University, St. Patrick’s Campus, Drumcondra, Dublin 9, Ireland; 2 UCD School of Biosystems and Food Engineering, University College Dublin, Belfield, Dublin 4, Ireland

**Keywords:** Remote sensing, Peatlands, Drain detection, Carbon dynamics, Satellite imagery, GIS/Object based image analysis, Cost effective, Ecosystem services

## Abstract

**Background:**

Peatlands play an important role in the global carbon cycle. They provide important ecosystem services including carbon sequestration and storage. Drainage disturbs peatland ecosystem services. Mapping drains is difficult and expensive and their spatial extent is, in many cases, unknown. An object based image analysis (OBIA) was performed on a very high resolution satellite image (Geoeye-1) to extract information about drain location and extent on a blanket peatland in Ireland. Two accuracy assessment methods: *Error matrix* and the *completeness, correctness and quality* (*CCQ*) were used to assess the extracted data across the peatland and at several sub sites. The cost of the OBIA method was compared with manual digitisation and field survey. The drain maps were also used to assess the costs relating to blocking drains vs. a business-as-usual scenario and estimating the impact of each on carbon fluxes at the study site.

**Results:**

The OBIA method performed well at almost all sites. Almost 500 km of drains were detected within the peatland. In the *error matrix* method, overall accuracy (OA) of detecting the drains was 94% and the kappa statistic was 0.66. The OA for all sub-areas, except one, was 95–97%. The *CCQ* was 85%, 85% and 71% respectively. The OBIA method was the most cost effective way to map peatland drains and was at least 55% cheaper than either field survey or manual digitisation, respectively. The extracted drain maps were used constrain the study area CO_2_ flux which was 19% smaller than the prescribed Peatland Code value for drained peatlands.

**Conclusions:**

The OBIA method used in this study showed that it is possible to accurately extract maps of fine scale peatland drains over large areas in a cost effective manner. The development of methods to map the spatial extent of drains is important as they play a critical role in peatland carbon dynamics. The objective of this study was to extract data on the spatial extent of drains on a blanket bog in the west of Ireland. The results show that information on drain extent and location can be extracted from high resolution imagery and mapped with a high degree of accuracy. Under Article 3.4 of the Kyoto Protocol Annex 1 parties can account for greenhouse gas emission by sources and removals by sinks resulting from “wetlands drainage and rewetting”. The ability to map the spatial extent, density and location of peatlands drains means that Annex 1 parties can develop strategies for drain blocking to aid reduction of CO_2_ emissions, DOC runoff and water discoloration. This paper highlights some uncertainty around using one-size-fits-all emission factors for GHG in drained peatlands and re-wetting scenarios. However, the OBIA method is robust and accurate and could be used to assess the extent of drains in peatlands across the globe aiding the refinement of peatland carbon dynamics .

## Background

Peatlands represent about 2–3% of the global terrestrial environment and store about 25% of global soil organic carbon (SOC) stock [1], estimated to be 547 Gt [2]. On an a per-area basis, peatlands store more carbon than any other terrestrial ecosystem [3], but this carbon stock is vulnerable to ecosystem disturbance [4–8]. Disturbance can be natural or anthropogenic [9] and includes drainage to enable the development of agriculture, forestry, peat extraction (for fuel or horticulture) or for road construction [8, 10, 11]. Drainage is the first step in the anthropogenic modification of peatlands [12–15]. Peatlands in both the southern and northern hemispheres have been drained [12, 14, 16], and between 1990 and 2008, global CO_2_ emissions from drained peatland increased by about 20% from 1058 to 1298 Mton [17]. The drainage and conversion of about 308,500 km^2^ or 52% of Europe’s temperate bogs for peat mining and agriculture over the last century has turned them from a moderate sink to a source of greenhouse gases [18, 19].

Much research has focused on the hydrological and physical effects of drainage on peatland ecosystems, but in recent years, there has been a concerted effort to understand the effects of drainage and subsequent drain blocking on ecosystem functions and dissolved organic carbon (DOC) dynamics, particulate organic carbon (POC) dynamics and net C flux [11, 16, 20–25]. Open cut drains increase drainage density and lower the water table of peatlands [22]. They can also lead to drying, shrinkage and subsidence in the surrounding peat. In Wales, the cutting of drains into upland blanket bog led to the creation of localised dry zones within 2 m of the drains, a reduction in surface water and a lowering of the water table within 5 m of the drains [25]. Such modifications change the balance between aerobic and anaerobic conditions in the peatland, exposing anaerobic zones to oxygen and increased oxidisation [24]. Peatland drains are greenhouse gas hotspots [26, 27], they also act as conduits allowing DOC and POC to be released into the natural drainage network, discolouring drinking water supplies and contributing to C emissions [22, 26, 28–30]. The SOC stock of peatlands is stable only so long as water-logged anaerobic conditions are maintained [31, 32]. Therefore drainage can lead to mobilisation of carbon via CO_2,_ DOC and POC as well as increasing the risk of deeper peat drying and becoming more vulnerable to fire events [8].

With new understandings of peatlands and their importance as stores of carbon (C), earlier attempts to drain peatlands are now regarded as being a threat to the preservation of the ecosystem and its C security [30, 33]. Given the potential impact that drains have on peatland ecosystem services there is a need to identify drained networks for conservation assessment [34]. However, determining the extent, location and density of drainage systems is often a difficult and expensive task [22]. Peatland drains have been surveyed in several upland blanket peat catchments in northern Britain [22] and have been mapped using ground surveys and aerial photography interpretation [20, 35]. Airborne thermal imagery and LiDAR have been used to measure near surface hydrology but these data underestimated the volume and depth of surface drainage networks [33]. Field surveys produce accurate datasets however, they involve high labour costs [34, 36]. Therefore, the task of determining the extent, location and density of drainage systems is often difficult and expensive [22].

The extent of peatland drains can clearly be seen on high resolution imagery [37], therefore image analysis methods to extract these data could be used to create drain maps. Remote sensing can be used to map and monitor peatlands over very wide areas [38] using both spectral-oriented data [39, 40] and object-oriented data [41]. Low and medium resolution multispectral imagery have been used to map the extent of peatlands. Pflugmacher et al. [40] examined the potential of using the moderate resolution imaging spectroradiometer (MODIS) low resolution imagery to map peatlands over large areas. Medium resolution satellite imagery has also been used to map peatlands on the Isle of Skye in Scotland [42]. MODIS (250 m resolution) was also used to examine peatland disturbance in the Wicklow Mountains [43]. However, while low and medium resolution satellite images are useful for broad scale mapping of peatland extent and condition over large areas [44], only high resolution imagery is suitable for mapping sub-metre sized linear features such as drains.

Object based image analysis (OBIA) software can be trained to extract the spatial extent of specific objects or features from medium and high resolution images using machine learning techniques with user-defined spectral, spatial, temporal and ancillary information [35, 45]. Peatlands in Quebec, Canada, were classified using an object-oriented approach with SPOT-4 imagery [46]. An object-oriented classification was used with medium resolution India Remote Sensing (IRS) imagery to identify disturbance on raised bogs in Ireland [44]. A semi-automatic object based approach was used to map the extent of peatlands in James Bay, Quebec, Canada very high resolution QuickBird imagery [47]. Evrendilek et al. [38] used Geoeye-1 imagery to quantify changes in a peatland between 1944 and 2009, although they had difficulty separating water bodies and ditches.

Peatland drains are linear features that are both small (i.e. less than 2 m wide) and spatially extensive [48]. OBIA is a useful tool for extracting fine scale features from high resolution imagery [44]. Extracted data can be used to refine estimates of the impact of anthropogenic disturbance on peatlands and their carbon stocks. There is a lack of information on the development of techniques to extract fine scale drain data from peatlands. However, OBIA has been used to extract fine scale linear features such as roads, power line tracks and informal walking trails. Narrow linear disturbance features have been detected in forests used both very high resolution (VHR) QuickBird and medium resolution SPOT imagery [49] and object-based image analysis has been used to extract roads from a variety of backgrounds [50].

The objective of this work was to extract information on the extent of man-made drains in a peatland area using OBIA and high resolution imagery. As drains impact on the carbon dynamics and ecosystem function of peatlands, this technique could be a cost-effective way to identify and map the spatial extent of peatland drains over large area to refine land use and management plans.

## Methods

**Fig. 1:**
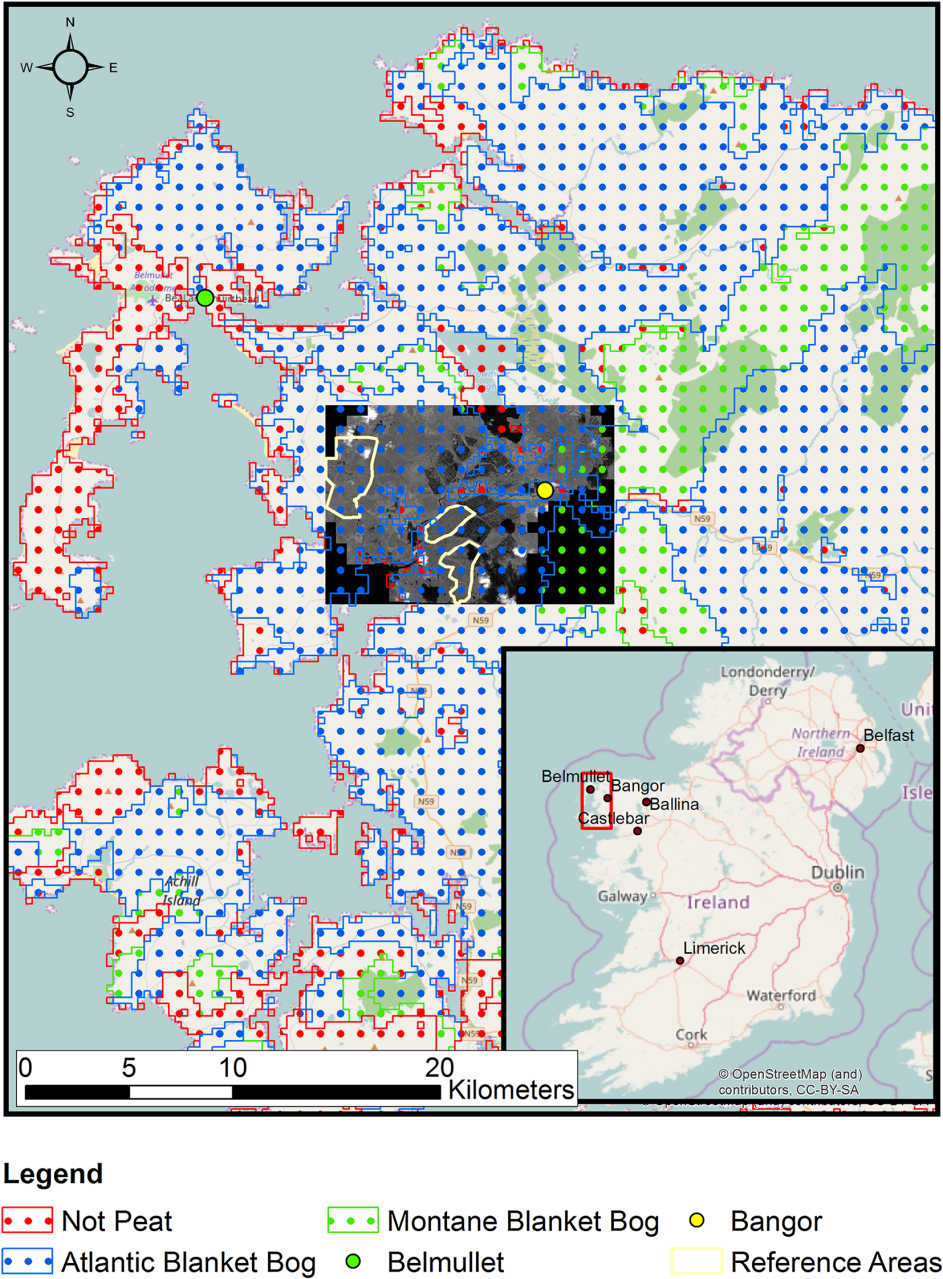
Location of the study area, Co. Mayo, Ireland

The study area was located within a low-level Atlantic blanket bog complex in the West of Ireland [51] (54°7′41″N, 9°48′44″W) (Fig. 1). The area of this peatland is ca. 11,750 ha and it is located close to sea level (from sea level to ~70 m asl). The mean (1961–1990) annual precipitation at the nearby Belmullet weather station was 1142 mm [52]. Peat depth is estimated to range from 0.04 to 0.58 m [53]. Peat depth observations from the nearby Glenamoy bog-complex indicated this range is realistic [54]. An area of about 1700 ha [55] was drained in the late 1950s (David Fallon, Personal Communication) and cutaway to provide fuel for the nearby de-commissioned Bellacorrick peat-fired power station [13]. Much of this industrial peatland area was exhausted (referred to as cutaway) and taken out of production [56]. Several other areas comprising of ~1203 ha of Atlantic blanket bog had small surface drains (called ditches) installed. The peat at these sites was not extracted but the drains were not blocked. Intact blanket bog in the area has been designated EU Priority Habitat status under the Habitats directive 92/43/EEC [56].

A geo- and ortho- rectified multi-spectral image (*Geoeye*-*1*) was acquired for the study area on the 26th August 2010 GMT. The image covered 68 km^2^. Geoeye-1 has four spectral bands: blue (450–510 nm); green (510–580 nm); red (655–690 nm); near infra-red (780–920 nm). These spectral bands have a spatial resolution of 1.84 m [57]. The image also has a higher resolution panchromatic band (0.46 m) with a spectral range from 450–800 nm. In the image there is 7% cloud cover and the local solar azimuth angle was 164.97° and the local solar zenith angle was 45.46°. The multispectral image was pan-sharpened to 0.5 m using the Brovey transform method in ArcGIS to create the very high-resolution image [58] which was used to aid identification of the extent of peatland drains. Within the area of interest the drains were straight and about 1 m wide. They are located at intervals of 15 m. However, there were some instances where the side walls of drains have collapsed or they have infilled with vegetation.

The first step for the OBIA was to delineate the extent of peatland in the image (*Geoeye*-*1*) using the Derived Irish Peat Map Version 2 (DIPMv2) [51]. It was assumed that all areas falling inside the DIPMv2 delineated area were peat. Urban areas, lakes, agricultural land, clouds and shadows were all masked. A training dataset was created by digitising selected linear drains in the image. Most of the drains that were digitised are intact and therefore easy to see in the image. However, some drains have collapsed or have are overgrown with vegetation. These drains were also digitised. In the digitisation process a line was drawn along the centre of each drain. This helps to ensure that spectral contamination from edge pixels was minimised. Digitised segment lengths ranged from 0.34 to 434 m with an average length of 100 m. The resulting training dataset consists of 700 digitised lines extending to 70,263 m.

These training data were used to train the OBIA software [*Feature Analyst* (FA)] to identify and extract peatland drains. In FA, the OBIA algorithms are proprietary and the training is implemented in a black box. However, FA uses spatial context information, meaning that some parameters can be adjusted by the user at the start of the extraction process [59]. Here, several FA spatial parameters were selected and adjusted including: *Feature Selector*—Small Manmade Feature which was set at <5 m; *Input Representation*—the Bull’s Eye 1 option with Pattern Width 11 was selected; *Masking* included limiting the OBIA to the study area (type 1) as well as including both a cloud and shadow mask. The output options that were selected included *output to vector* and the *aggregation* of a minimum area of 0.25 m^2^ [59]. The output from the FA process is a vector file of linear features depicting an extensive network of drainage ditches. Closer examination revealed many errors in the drains extraction vector file. Landscape features including small steep slopes, shadow and streams were included. A key feature of FA is the iterative correction function. This allows for manual inspection of the output features and identification of both correct and incorrect features. Once this advanced training is complete the software can be re-run, refining the results. This iterative process removed erroneous classifications enabling the development of more accurate maps of the peatlands drains.

The traditional method for assessing the accuracy of maps is to use an error matrix (EM) [60]. The EM enables the assessment of four variables: (1) true positives (drains in the image and on the ground); (2) true negatives (not drains in the image and on the ground); (3) false positives (not drains on the ground but drains in the image); and (4) false negatives (drains on the ground but not drains in the image). These data can then be used to calculate the user’s accuracy (UA); producer’s accuracy (PA); overall accuracy (OA) [60] and kappa statistic (KS) [61]. Given that the EM method is usually applied to assessing area it was decided to use a second assessment method which had been designed for assessing linear features such as roads i.e. the completeness, correctness and quality (CCQ) method [62].

**Fig. 2:**
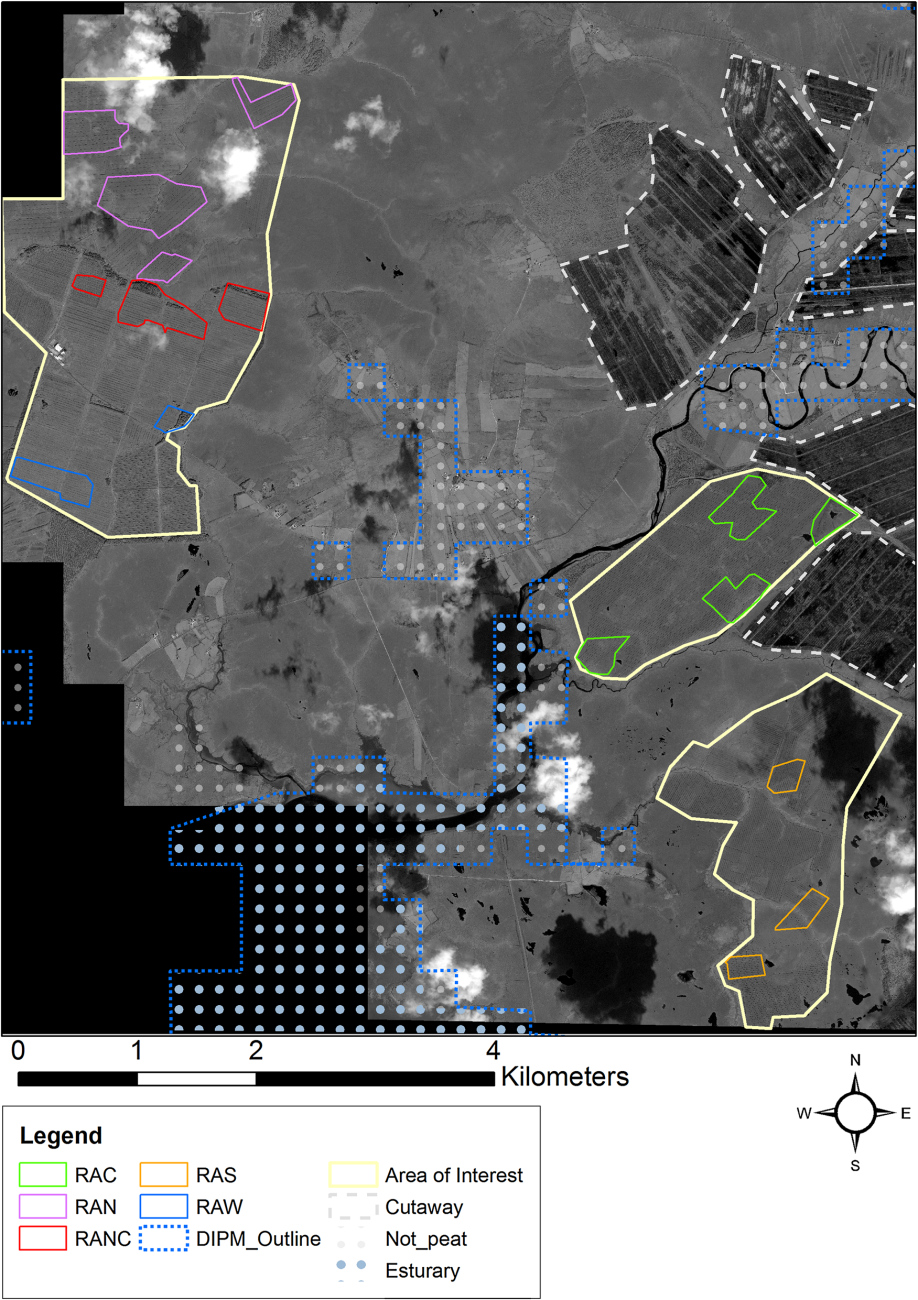
The reference areas within the study site

Within the study area, five sub areas were delineated as reference areas i.e. reference area North (RAN); reference area North Central (RANC); reference area West (RAW); reference area Central (RAC) and reference area South (RAS) (Fig. 2). The first step in the EM method is to buffer the output map linear features to 1.5 m. This buffer gives each line an area which represents the width of the drains on the ground. Hawth’s tools [63] (an extension for ArcGIS) was used to create and randomly distribute 3000 data points across all five reference areas. In addition, 500 independent data points were created and randomly distributed within each reference area. This dual approach allowed the assessment of accuracy across the entire area as well as assessment within each reference area. Points that fall within the buffered areas were classified as a drain; those outside the buffered areas were classified as not-drains.

There were some issues with the EM method: the buffered drains cover a very small percentage area of the entire area. Most of the random points fell outside these buffered areas, in the not-drain areas. The reference data was therefore biased towards these areas. While the EM results depict a relatively good accuracy, the authors felt it was necessary to use an accuracy assessment method that specifically examines the linear features themselves.

The CCQ has been used to assess the accuracy of extracted data related to roads [62]. However, CCQ requires a comprehensive validation dataset. This was created by manually digitising all drains within each reference area resulting in reference dataset extending to 76,635 m (minimum and maximum segment lengths of 0.35–435 m). In the CCQ method, the output vector file is compared to this high-quality validation dataset. The accuracy of how well the extracted dataset relates to the reference dataset [62, 64] is examined. Completeness refers to the percentage of the reference network that is successfully extracted by the detection algorithm, correctness is the percentage of extracted network matched by the reference network and quality is the contribution of the matched roads to the entire extracted and reference network [64]. Higher percentage values indicate more accurate results.

The cost effectiveness of using OBIA versus manual digitisation and field survey was also examined. The main variables included were labour, data, software costs and equipment needed for the field survey. The labour costs associated with both the manual digitisation (MD) and OBIA methods are similar i.e. labour = €17/h (according to the Irish University Association (IUA) rates for a research assistant); imagery and software coasts (€2000). However, some ground truthing is essential for the OBIA method and requires a surveyor and field assistant (€44, €14/h respectively) as well as factoring in mileage, car hire and accommodation costs. The costs associated with the field survey include health and safety in the field i.e. it is necessary to have two people working together in the field thus labour is more expensive i.e. €44/h (William Hamilton, Personal Communication) for an experienced surveyor and €14/h (IUA rates) for a field assistant. There are also various field survey equipment and costs including high accuracy GPS, travel and accommodation (€3520).

The effect of the drains on the carbon dioxide (CO_2_), Methane (CH_4_) and DOC fluxes at the study site was calculated for six scenarios using different emission factors from several studies in the Ireland, the UK and from the IPCC (Table 1). The two intact blanket bog scenarios are included as controls. The Fluxes for intact blanket bog A are calculated using figures from Koehler et al. [65] for a blanket bog in south-west Ireland. While intact blanket bog B uses IPCC Tier 1 figures for CO_2_ and CH_4_ and the Koehler et al. figure [65] for DOC. There are two drained scenarios: the Drained/BAU (DBAU) scenario uses emission factors from Reed et al. [66] and Evans et al. [26], while the Mapped Drains/BAU (MDBAU) scenario uses the drain area extracted in this study with figures for CO_2_ from Wilson et al. [67, 68] (Table 2). Two re-wetting scenarios are included used IPCC [69, 70] and local figures [65, 71] for the various fluxes and used to examine the different between BAU and re-wetting.

**Table 1:** Emission Factors for six scenarios (t CO_2_ eq. ha^−1^ year^−1^) and study area fluxes (t CO_2_ eq.) with estimated costs of re-wetting vs business as usual

	Intact Blanket bogA^c^	Intact Blanket bogB^d^	DBAU^a, e^	MDBAU^b, f^	Re-wetA^g^	Re-wetB^h^
CO_2_	−0.48	−2.12	1.40	1.14	0.04	−1.04
CH_4_	0.04	1.73	2.00	2.00	1.73	0.09
DOC	0.14	0.14	1.00	1.00	0.14	0.14
Emission factor	−0.30	−0.25	4.40	4.14	1.91	−0.81
Study area (t CO_2_ eq.)	−357	−301	5294	4984	2298	−975
EU ETS rate: €5.48^i^						
Drain blocking^j^	€0	€0	€0	€0	€486,100	€486,100
Annual emission costs	−€1,958^k^	−€1648	€29,010	€27,312	€12,593	−€5340
Initial cost (€) year 1	−€1958	−€1648	€29,010	€27,312	€498,693	€480,759
Total costs (year 30)^l^	−€58,744	−€49,448	€870,288	€819,356	€863,884	€325,888

^a^Drained/business as usual

^b^Mapped drains/ business as usual

^c^CO_2_, CH_4_ and DOC derived from Koehler et al. [65]

^d^CO_2_ derived from Bonn et al. [69] and CH_4_ IPCC Tier 1 [70] and DOC from Koehler et al. [65]

^e^CO_2_ and CH_4_ derived from Reed et al. [66] and DOC from Evans et al. [26]

^f^CO_2_ derived in this study using figures from Wilson et al. [67, 68], CH_4_ Reed et al. [66] and DOC Evans et al. [26]

^g^CO_2_ and CH_4_ from Bonn et al. [69] and IPCC Tier 1. [70] and DOC from Koehler [65]

^h^CO_2_ and CH_4_ from Wilson et al. [71] and DOC from Koehler et al. [65]

^i^€5.48 per tonne CO_2_ (21/11/2016—https://carbon-pulse.com/category/eu-ets/)

^j^Includes the cost of OBIA (€4852) and cost of blocking drains (€481,247) at €400/ha*1203 ha

^k^The minus value indicates the amount that is saved by not draining peatlands

^l^The total costs equals the initial year 1 cost plus the annual emission cost multiplied by 29

**Table 2:** Using drain extent extracted here to estimate CO_2_ emissions in the drains and adjacent zones

	Area (ha)	t CO_2_ eq. ha^−1^ year^−1^	Study site t CO_2_ eq.
Drain^a^	68	0.53	36.16
Drain +2 m^b^ (zone 1)	199	1.76	350.67
Drain 2 m +3 m^b ^(zone 2)	239	1.14	272.36
Drain 5 m +1.5 m^b^ (zone 3)	697	1.14	794.28
Average site emissions		1.14	
Total	1203		1453.47

^a^Wilson et al. [67]

^b^Wilson et al. [68]

In the mapped drain scenario, the peatland was divided into zones based on the Wilson et al. [11] observations that drains lead to localised dry zones within 2 m of drains, and a reduction in surface water and a lowering of the water table within 5 m of the drains [25]. This information was used to segregate the peatland into several zones. The drain polygons extracted in the OBIA method are about 1.4 m wide. The area of these drain polygons was calculated using the OBIA extracted drains (see Table 2). They were buffered using tools in ArcGIS 10.2, to represent the dryer zones identified by Wilson et al. [11]. These zones included: (1) localised dry zones (the drain area was buffered to 2 m in ArcGIS); (2) lower water table zones (the localised dry zone areas were buffered to 3 m) and (3) the remaining peat located outside these buffered zones was, in most cases, about 3 m wide. Each zone was assigned an emission value (Table 2) and it was assumed zone 3 had a similar value to zone 2 as the high drainage density prohibited wetter areas there.

Estimated costs and potential savings were calculated for the scenarios using the EU Emissions Trading Scheme (ETS) rates for CO_2_ [72]. The costs for both the intact and drained/BAU scenarios were based on the total study area flux multiplied by the EU-ETS rate on 21st November, 2016. The re-wetting scenarios included the cost of OBIA and drain blocking. Bord na Móna calculated that the average cost of blocking drains on a raised bog was €400/ha [73], although others in the UK have found that figure to be higher [74]. All values were calculated for a thirty-year period [66].

## Results

**Fig. 3:**
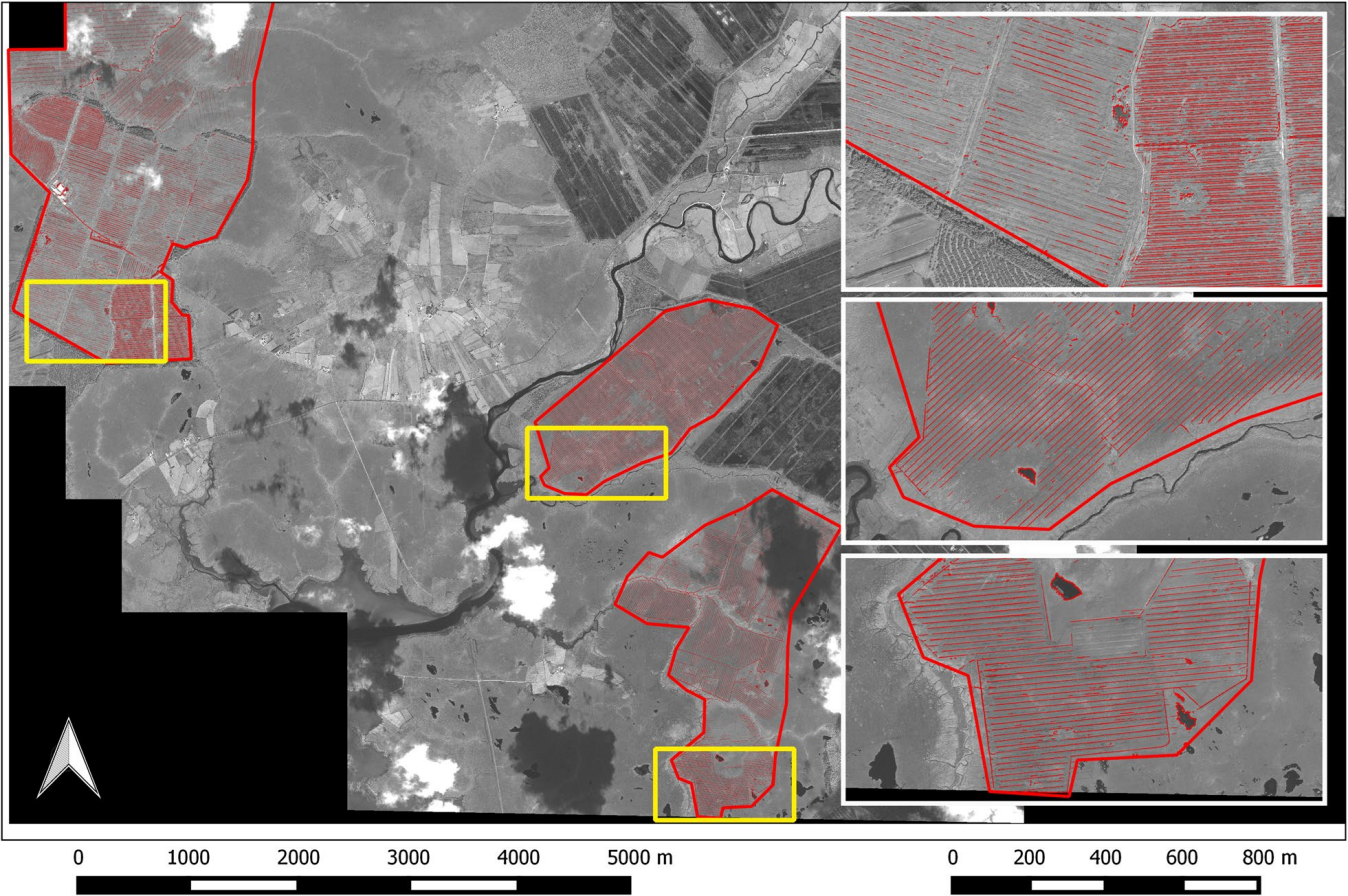
Spatial extent of extracted drains within the study area

Within the peatland complex 496,630 m of drains were identified (Fig. 3). The first iteration identified many non-man made features such as rivers, streams and shadows in forestry plantations. The second iteration of the OBIA process removed these features and on further analysis resulted in the most accurate drain maps. An initial visual analysis of the results indicated that the feature extraction process had performed well in extracting the extent of drains. This was supported by quantitative analysis (Table 3). The EM method reported the overall accuracy for the extracted drains across the entire reference area to be 94%, the user’s accuracy was 83% and the producer’s accuracy was 60%. These results indicate that from a map user’s point of view the method worked well. This was supported by a kappa statistic of 0.66 which shows substantial agreement [61, 75] between the extracted data and the reference data. The CCQ also depicts a high accuracy of the OBIA method for detecting the drains in this area. Across the entire study area, the completeness = 85%, correctness = 85% and quality = 71%. This indicates that 85% of the reference dataset was identified by the extracted drain data, that 85% of the extracted drains were matched by the reference data and 71% of the matched drains contributed to the entire extraction and reference drain network.

**Table 3:** The error matrix for accuracy assessment of the reference areas within the study area

Extracted peatland drains					
Linear validation dataset	Drain	No drain		Total	Producer accuracy
Drain	193	131		324	0.60
No drain	39	2637		2676	0.99
			2830		
Total	232	2768		3000	
User accuracy	0.83	0.95			
Kappa statistic	0.66			OA	0.94

Within the five reference areas, the EM method showed that there was little variation in overall accuracy and producer’s accuracy for *not*-*drain*, but some variation in the producer’s accuracy, user’s accuracy and Kappa Statistics for the *drains*. This variation was also reflected in the CCQ data (Table 4). The greater values for producer’s accuracy and user’s accuracy for not drained pixels indicate little misclassification but this seemed to be less reliable for the drain pixels. Of particular note is the producer’s accuracy for the RAW which was <50% indicating some misclassification. This low value is also seen in the CCQ assessment. The extraction method works well for detecting drainage in this peatland area located in the West of Ireland. It works particularly well for detecting drains that were clearly visible by eye in the high-resolution imagery. However, it was also reliable for detecting drains that have experience some level of change i.e. have collapsed or are overgrown.

**Table 4:** Error matrices and CCQ results for overall and five sub reference areas

Name	OA (%)	PA		UA		KS (%)	Completeness (%)	Correctness (%)	Quality (%)
		D (%)	ND (%)	D (%)	ND (%)				
RAN	95	61	99	88	96	70	81	93	75
RANC	95	67	99	88	96	73	87	84	74
RAW	93	43	100	92	93	92	74	81	59
RAC	97	82	98	87	98	83	92	92	85
RAS	95	74	97	77	97	73	86	83	73
AOI	94	60	99	83	95	66	85	85	71

*D* drain, *ND* not drain, *OA* overall assessment, *PA* producer’s accuracy, *UA* user’s accuracy, *KS* kappa statistic, *AOI* area of interest i.e. total study site

In terms of cost effectiveness, the OBIA method was estimated to be the least expensive way of mapping peatland drains. It was considerably cheaper than manual digitisation and field survey, both of which are relatively labour intensive (Table 5) or require high additional costs (Equipment, travel, accommodation). The estimated cost for mapping the drains at this study site was €4852 (OBIA); €10,690 (field survey) and €11,459 (manual digitisation). The flux scenarios for this site ranged from −357 t CO_2_ eq. to 5294 t CO_2_ eq. for the intact bog A and DBAU scenarios, respectively. The costs for each scenario can be seen in Table 1.

**Table 5:** Comparison cost of each drain assessment method

Methods		Amount	Rate (€)	Total cost (€)
Manual digitisation	Labour (hours)	583	17^a^	9951
	Imagery	1	1256	1256
	Software cost (per day)	97	2.6^b^	252
	Total			11,459
OBIA	Image analysis labour (hours)	120	17^a^	2047
	Imagery	1	1265	1265
	Software cost (per day)	20	4.1^c^	82
	Surveyor labour (hours)	16	44	700
	Field assistant labour (hours)	16	14	224
	Equipment (per job)	–	–	19
	Lodgings and subsistence	1	125	125
	Mileage (km)	576	0.59	340
	Car hire	1	50^d^	50
	Total			4852
Field survey	Labour surveyor (hours)	124.2	44	5432
	Labour field assistant (hours)	124.2	14	1738
	Equipment (per job)	–	–	144
	Lodgings and subsistence	16	125	2000
	Mileage (km)	1060	0.59	626
	Car hire	1	750^e^	750
	Total			10,690

^a^ €17/h is calculated from the Irish Universities Association research pay scale for a research assistant on point 10 of the salary scale working

^b, c^Software cost per day (assuming a 3 year depreciation and the number of workdays as a ratio of 3 years of workdays as the proportion to cost to the calculation e.g. if used for 15 days, then [15 / (260 × 3)]*2000] = cost of software)

^d, e^Car hire for 2 and 16 days, respectively

## Discussion

According to both the EM and CCQ methods, the OBIA performs well detecting ~500 km of drains across the study area. It was also the most cost effect way of mapping peatland drains in this intensively drained peatland area. In 2010, the condition of the drains ranged from intact to collapsed or overgrown. Intact drains were clearly visible while the collapsed/overgrown drains were not. Each sub-area was analysed individually to examine the effect of drain condition on accuracy. In the reference areas where the drains were in good condition (RAC, RAN, RAS and RANC) the OBIA method performed well (Table 4). The accurate extraction of drains was likely due to the high spectral contrast between dark peaty water in the drains and lighter vegetation on the surrounding peatland. However, as the drain condition deteriorates e.g. in the RAW area, vegetation invades the drains and the spectral contrast diminishes. This affects the ability of the OBIA method to detect these drains. The result of this could be seen in both the EM and CCQ accuracy assessment methods for RAW (Table 4). However, despite the reduced accuracy the method does extract the general structure and pattern of drains in the RAW area.

Map outputs need to be assessed for accuracy. Traditionally, when assessing the accuracy of a spatial area, an error matrix was used to assess user, producer and overall accuracy [60]. However there may be bias in its use in relation to the assessment of linear features [76]. Drains are usually represented as lines on a map. Linear features can be extensive over a spatial area, as in this case, they do not however have an area. The discretely sampled points used to populate an error matrix may be biased towards one land use because of the way it is represented on a map i.e. a drain (linear feature). In this case, the examination of accuracy involved a binary decision, either it is a drain or it is not a drain. Since the non-drain polygons cover much larger areas of land compared to the area covered by linear drain features, there was a strong bias towards non-drain polygons in the random point data. This can be seen in Table 4, where a very large number of random sample points (3000) were needed to ensure that the drains were adequately represented in the sample (324 or ~10%). This issue was somewhat alleviated by buffering the drains to give them an area (this area represents the drain on the ground more accurately than a line can). Despite the extraction method performing well in the EM method, it was felt that there was an optimistic bias in the data because about 90% of the sample points were restricted to homogenous non-drain areas [77]. Lathrop et al. [76] found that the EM accuracy assessment method, using discrete sample points, did not work well with linear object features because points are examined as coincident pixels rather than the objects [78]. Therefore, another method was needed to assess the accuracy of the OBIA method more reliably. The CCQ method overcame this optimistic bias. It measures how well the extracted data overlaps with a reference dataset. Overall, the CCQ method delivered a better assessment of the accuracy of the extracted data as it was assessed solely in relation to the high-quality reference dataset and there was no bias towards large homogenous areas i.e. non-drain.

This OBIA approach enabled a relatively rapid assessment of drain density at this site. Both MD and field survey are possible, however, MD was estimated to take over six times longer and was almost three time more expensive (Table 5). A field survey would take a similar amount of time to OBIA but was more than twice as expensive due to the safety requirement of having two people working in the field. While the OBIA method was not fully is automated, it did enable drain density to be accurately and cost effectively mapped over large areas. This OBIA method created accurate maps that record the spatial extent and drainage density of peatland drains. The condition of drains can also be inferred i.e. where the accuracy is lower and a drainage pattern is extracted it may indicate drains that are overgrown.

The method is robust and relatively easy to implement and could aid in the estimation of the impact of Land use, land use change and forestry in a cost-effective way. Emissions from Land Use Land Use Change and Forestry (LULUCF) are currently not accounted for in internal EU targets, they will, however be included in the EU’s 2nd commitment period target in the Kyoto Protocol [79]. Since 2013, the Kyoto Protocol has allowed Annex 1 parties to account for greenhouse gas emission by sources and removals by sinks resulting from Wetlands Drainage and Rewetting (WDR) under Article 3.4. [80]. In the light of the Paris Agreement; EU targets for the 2nd commitment period and the Mapping and Assessment of Ecosystem Services (MAES) initiative [81] it is necessary to begin to refine spatial data related landscape carbon dynamics.

Wilson et al. [71] suggest that the one-size fits all approach may not be appropriate for different sites, particularly in relation to drain extent and its impact on the study area emissions. The main difference between the DBAU and MDBAU is related to the how the CO_2_ figure is calculated. The DBAU scenario is estimated on a tonnes of CO_2_ per hectare value derived from the Peatland Code [82]. While in MDBAU scenario, the area of drains and zones adjacent to drains is extracted using the OBIA method using flux measurements for each zone (Table 2). The emission factor for CO_2_ in the MDBAU scenario is about 19% lower that then the Peatland Code value. This difference equals to a €50,000 reduction in costs over the 30-year period for this site of 1203 ha. When comparing both BAU scenarios with the re-wet scenarios, it is clear that the uncertainty between the IPCC Tier 1 and local emission factors have large implications with regard to the costs reduction. Analysis of these issues is beyond the scope of this paper, and it is difficult to come to a decisive conclusion without further clarification and refinement.

In this study, the OBIA method is successful in extracting maps of drainage extent and density from the Geoeye-1 high-resolution satellite image. Peatlands provide a number of ecosystem services including water purification and carbon storage [10, 83]. Drains impact on these services enhancing both emissions of CO_2_ and removal of DOC [30, 84]. This method could be used in tropical areas, though its use may be limited in areas where trees have overgrown and blocked the drain spectral signal. In several areas of the study site and particularly in the RAW area, the drains are overgrown and infilled; despite this the general structure of the drains was identified. The method could be applied in temperate and boreal zones where peatlands have been drained. The identification and mapping of drains over large areas is a cost-effective aid for the management of drain blocking campaigns. With the issues related to the impact of drains on peatlands, peatland ecosystems services and Article 3.4, it is essential that accurate maps of artificial peatland drain systems can be produced to identify: 1. hotspots for CO_2_ emissions and DOC production and 2. suitable areas for drain blocking and rewetting.

Satellite remote sensing is a good tool for mapping peatland disturbance. While coarse spatial resolution imagery can be useful for mapping broad scale disturbance [43, 85], high resolution imagery is essential for mapping peatland drains [44, 68]. The Copernicus satellites including Sentinal-1 and Sentinel-2 provide free images at high spatial (10 m) and temporal resolutions [86]. However, to detect small linear features, such as the industrial scale drainage featured in this study or drains create for domestic peat extraction, it is necessary to acquire very high spatial resolution images such as GeoEye-1 satellite data or images from aerial or UAV platforms [18, 44]. The semi-automatic assessment methods used in this study can be used to extract valuable information useful to land managers and policy makers. OBIA offers a cost effective and accurate approach for extracting information on drainage extent and density from very high resolution satellite imagery. This semi-automatic method could be used to delineate and map drains and CO_2_ hotspots in large agricultural, peatland and wetland areas across the globe.

## Conclusions

The OBIA method used in this study shows that it is possible to accurately extract fine scale features such as peatland drains over large areas, producing cost-effective maps. Globally, peatlands have been drained to support production of crops, fuel, livestock and timber [26]. Peatland drainage is extensive and increases aerobic decomposition and DOC export leading to a loss of ecosystem services such as sequestration and storage of C and the potential development of greenhouse gas hotspots [8, 26, 27]. Despite the impact that drainage has on peatland carbon dynamics, there is a lack of methods for assessing the spatial extent and condition of peatland drains. Historically, this may be due to the difficulty and expense of surveying or mapping these small-scale features. However, as high resolution satellite data becomes increasingly available and software is developed to semi-automatically and automatically extract fine scale features, new insights into the carbon dynamics of natural ecosystems can be expected. The OBIA method explored here performed well for extracting data on the spatial extent and condition of peatland drains. The mapping of peatland drains is important in assessing how these extensive fine scale features may contribute to carbon dynamics in these sensitive ecosystems. This information is important for management, regulation and policy. The approach taken in this paper is robust and accurate. It could be applied to map the extent of drains aiding the monitoring of carbon dynamics in peatlands and wetlands across the globe.

## Abbreviations

asl: above sea level
C: carbon
CCQ: completeness, correctness and quality
CO_2_: carbon dioxide
DBAU: drained/business as usual
DIPMv2: derived Irish peat map version 2
DOC: dissolved organic carbon
EM: error matrix
EU: European Union
FA: feature analyst
Gt: gigaton
KS: kappa statistic
LULUCF: land use, land use change and forestry
MAES: mapping and assessment of ecosystem services
MDBAU: mapped drains/business as usual
MODIS: moderate resolution imaging spectroradiometer
Mton: megaton
OA: overall accuracy
OBIA: object based image analysis
PA: producer’s accuracy
POC: particulate organic carbon
rac: reference area Central
RAN: reference area North
RANC: reference area North Central
RAS: reference area South
RAW: reference area West
SOC: soil organic carbon
UA: user’s accuracy
VHR: very high resolution
